# Inhibition of IKK-*β* by epidioxysterols from the flowers of *Calotropis gigantea* (*Niu jiao gua*)

**DOI:** 10.1186/s13020-016-0081-1

**Published:** 2016-03-02

**Authors:** Supawadee Parhira, Guo-Yuan Zhu, Ting Li, Liang Liu, Li-Ping Bai, Zhi-Hong Jiang

**Affiliations:** State Key Laboratory of Quality Research in Chinese Medicine, Macau Institute for Applied Research in Medicine and Health, Macau University of Science and Technology, Taipa, Macau, China

## Abstract

**Background:**

*Calotropis gigantea* (Asclepiadaceae) (*Niu jiao gua*) has been used as a poultice in Chinese medicine for treating inflammatory skin diseases, e.g., neurodermatitis. This study aims to isolate the epidioxysterols from the flowers of *C. gigantea*, elucidate their structures and evaluate their possible inhibitory effects on the NF-*κ*B pathway.

**Methods:**

The two epidioxysterols 9,11-dehydroergosterol peroxide (**1**) and ergosterol peroxide (**2**) were isolated from the powdered flowers of *C. gigantea* by ultrasonic-assisted extraction, followed by the purification of the crude extract by column chromatography (i.e., silica gel and MCI-gel CHP 20P open columns). The chemical structures of these compounds were identified through a comparison of their HRMS, ^1^H and ^13^C NMR data with those in the literature. The in vitro IKK-*β* inhibitory activities of compounds **1** and **2** (1–100 µM) were evaluated using an IKK *α* and *β* Assay/Inhibitor Screening Kit, which is a single-site, semi-quantitative immunoassay. Berberine was used as a positive control. The IKK-*β* inhibitory activities between compounds **1** and **2** were compared by a two-tailed Student’s *t* test to summarize the structure activity relationship.

**Results:**

Compounds **1** and **2** exhibited a dose-dependent inhibitory activity towards IKK-*β* in a similar manner to that of berberine. The IKK-*β* inhibitory activities of these two epidioxysterols were significantly stronger (*P* = 0.001 for compound **1** and *P* = 0.028 for compound **2**) than that of berberine at the concentration of 100 µM. Furthermore, at the same concentration the suppressive effect of compound **1** towards IKK-*β* was greater than that of compound **2** (*P* = 0.041), while their activities at 10 and 50 µM were comparable. The difference in the results at 100 µM therefore suggested that the double bond between C-9 and C-11 in compound **1** could be responsible for its higher inhibitory activity towards IKK-*β* at this concentration.

**Conclusions:**

9,11-dehydroergosterol peroxide (**1**) and ergosterol peroxide (**2**) were isolated from the flowers of *C. gigantea* and exhibited in vitro inhibitory activities towards IKK-*β*.

**Electronic supplementary material:**

The online version of this article (doi:10.1186/s13020-016-0081-1) contains supplementary material, which is available to authorized users.

## Background

**Fig. 1:**
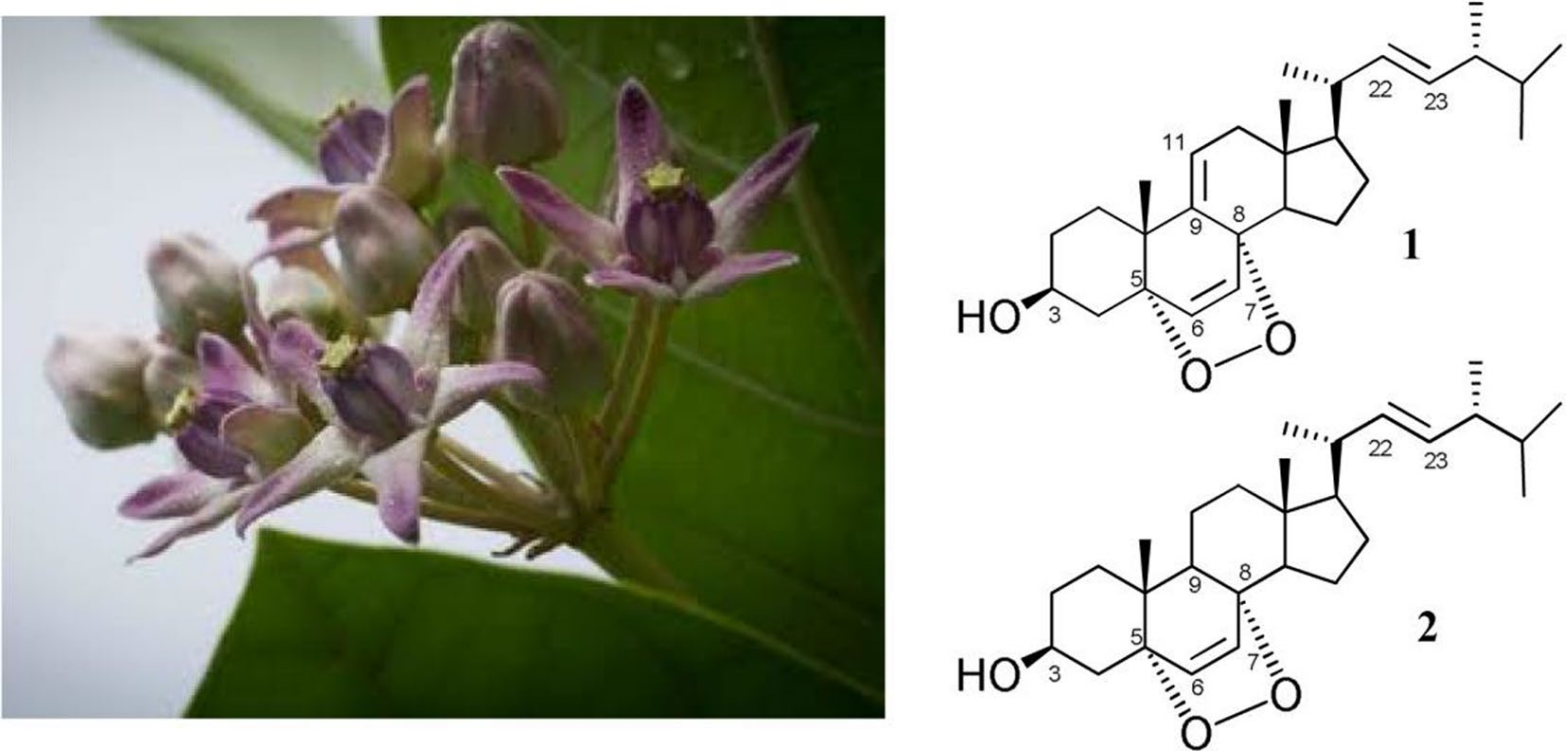
Flowers of *Calotropis gigantea* and chemical structures of compounds **1** and **2**

*Calotropis gigantea* (Asclepiadaceae; *Niu jiao gua*) (Fig. 1) has been used for centuries in Chinese medicine (CM) as a poultice for treating inflammatory skin diseases such as neurodermatitis [1]. Several preparations containing *C. gigantea*, such as “Swarna bhasma”, are also used for the treatment of several other inflammatory diseases, including asthma, rheumatoid arthritis and diabetes mellitus, in Ayurvedic medicine [2]. The ethyl acetate extract of the flowers of *C. gigantea* has been reported to exhibit antitumor activity against Ehrlich’s ascites carcinoma in vivo [3, 4]. A wide variety of bioactive compounds have been isolated from different parts of *C. gigantea*, including flavonoids [5], cardenolides [6, 7], sterols [8], pregnanone [9], triterpenes [10], non-protein amino acids [11] and lignan glycosides [12]. Among the various pharmacological activities reported for the compounds isolated from *C. gigantea*, their anticancer activities were the most significant of all [4–8].

The two epidioxysterols 9,11-dehydroergosterol peroxide (**1**) and ergosterol peroxide (**2**), which are shown in Fig. 1, are normally found in medicinal mushrooms. These compounds have been reported to exhibit a wide variety of pharmacological properties, including anti-inflammatory and anticancer activities [13–15]. It has been proposed that the anti-inflammatory activities of these compounds can be attributed to their inhibition of the nuclear factor kappa B (NF-*κ*B) pathway [13]. NF-*κ*B is involved in immune and inflammatory responses via the induction of pro-inflammatory genes. NF-*κ*B is associated with the regulatory protein I*κ*B*α* in the cytoplasm. Upon activation, I*κ*B*α* undergoes phosphorylation by the I*κ*B kinase (IKK) complex, followed by ubiquitination-dependent degradation. This degradation process leads to the nuclear translocation of NF-*κ*B to a specific DNA sequence, which results in the transcription of genes related to immune, inflammatory and tumorigenic responses [16–18]. IKK-*β*, which is an essential functional subunit of the IKK complex, is the key kinase responsible for the phosphorylation of I*κ*B*α*. The inhibition of IKK-*β* can therefore be used as an efficient strategy to block NF-*κ*B-mediated inflammatory responses. The discovery of new agents capable of inhibiting IKK-*β*, especially those from natural sources, could be useful for developing novel drugs for the treatment of inflammatory diseases and cancer.

It has been reported that compounds **1** and **2** can suppress LPS-induced inflammatory responses through their inhibition of the NF-*κ*B pathway, as well as inhibiting the growth of certain cancer cells, including HT29 (human colon adenocarcinoma cells), HL60 (human leukemia cells) and Hep 3B (human hepatocellular carcinoma cells) cells [13, 19, 20]. Moreover, compound **1** does not significantly inhibit the growth of WI38 cells (normal embryonic human fibroblasts) [19]. These studies therefore highlight the potential of these two promising epidioxysterols for the development of anti-inflammatory and anticancer agents.

The results of previous reports pertaining to the inhibitory effects of compounds **1** and **2** towards NF-*κ*B transcription with unknown molecular target [13–15] led us to investigate their in vitro IKK-*β* inhibitory activities with the aim of identifying their target. This study aims to isolate these two epidioxysterols from the flowers of *C. gigantea*, elucidate their structures and investigate their inhibitory effects towards the IKK-*β* enzyme.

## Methods

### Chemicals and reagents

Chloroform, methanol, petroleum ether (40‒60 °C) and acetone (AR grade) were purchased from RCI Labscan Limited (Bangkok, Thailand). Deuterated chloroform (CDCl_3_) for NMR analysis was purchased from Cambridge Isotope Laboratories, Inc. (Andover, MA, USA). Berberine was purchased from the National Institutes for Food and Drug Control of China (Beijing, China).

### General experimental procedures

The ^1^H and ^13^C NMR spectra were recorded on a Bruker Ascend 600 NMR spectrometer (Bruker, Karlsruhe, Germany; 600 and 150 MHz for the ^1^H and ^13^C NMR, respectively). All of the samples were prepared in CD_3_Cl and the chemical shifts of the solvent were used as internal references. The chemical shifts (*δ*) have been given in ppm, and the coupling constants (*J*) have been presented in Hz. High resolution mass spectrometry (HRMS) experiments were performed using an Agilent 6230 time-of-flight (TOF) mass spectrometer (Agilent, Santa Clara, CA, USA) with an atmospheric pressure chemical ionization (APCI) source. Purifications by column chromatography (CC) were performed over silica gel (40‒63 µm, Grace, Columbia, MD, USA) and MCI-gel CHP 20P (75‒150 µm, Mitsubishi Chemical Co. Ltd, Tokyo, Japan). Thin-layer chromatography (TLC) was conducted on pre-coated TLC Kieselgel 60 F_254_ plates (200 µm thick, Merck KGaA, Darmstadt, Germany). The TLC plates were visualized under ultraviolet (UV) light irradiation at a wavelength of 254 nm (Spectroline^®^, New York, NY, USA), as well as being sprayed with a 95 % EtOH solution containing 5 % sulfuric acid, followed by heating at 110 °C.

### Plant materials

Fresh *C. gigantea* flowers were purchased from a local market in Nakornprathom, Thailand during the months of October and November, 2010. The flowers were then air-dried at room temperature before being powdered in a blender. Each herbarium specimen was authenticated by comparison of morphological characteristics with an authenticated herbarium specimen of *C. gigantea* (No. MUST-CG201011), which was previously authenticated elsewhere [7, 12]. A voucher specimen of this plant (No. MUST–CG201012) was deposited at the State Key Laboratory of Quality Research in Chinese Medicine, Macau University of Science and Technology, China.

### Extraction and isolation

**Fig. 2:**
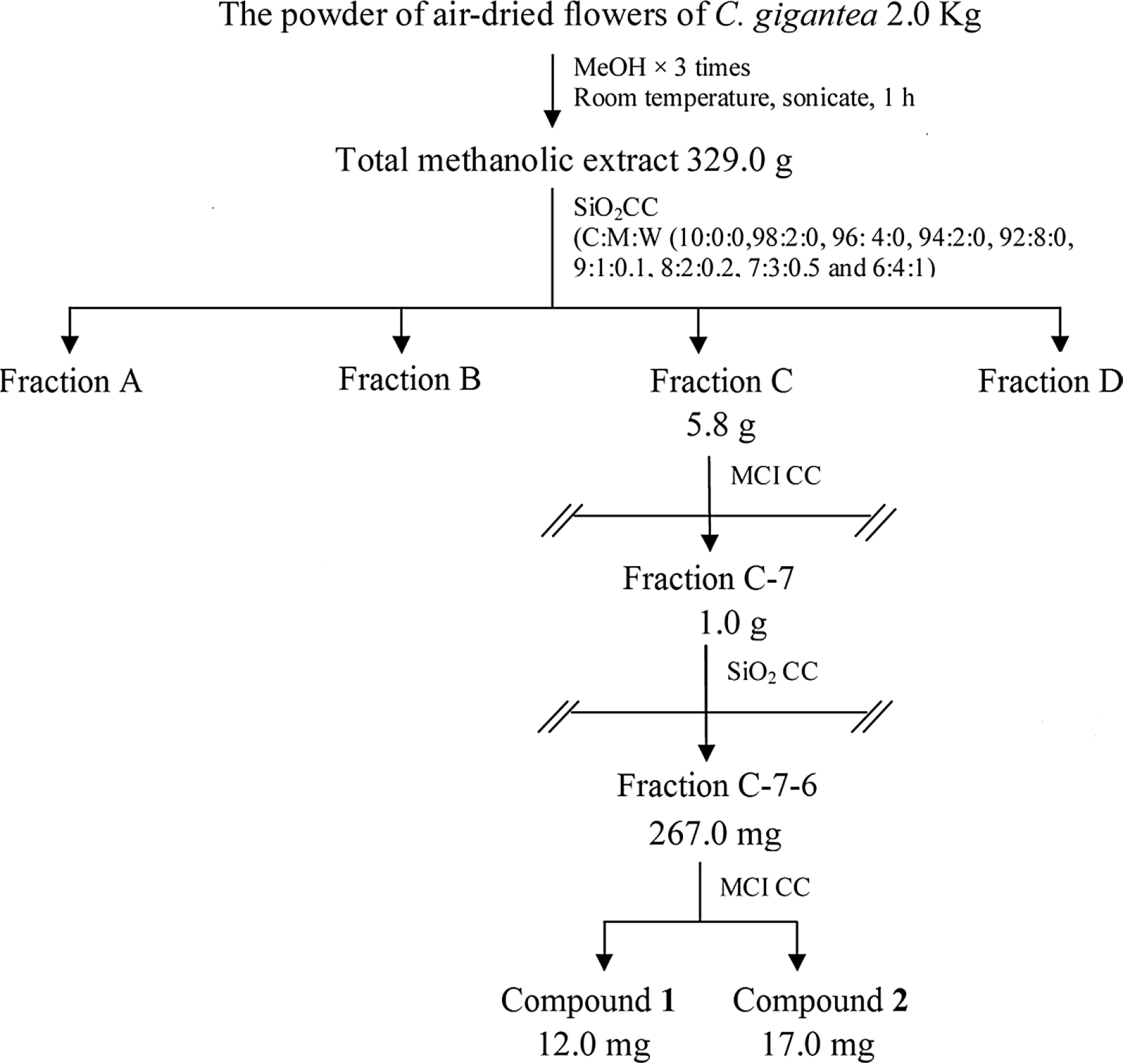
Schematic illustration of the isolation and purification of compounds **1** and **2**

The powdered air-dried flowers of *C. gigantea* (2.0 kg) were extracted with MeOH three times (1 h for each extraction) under ultrasonic using a Crest ultrasonic cleaner (Model 2800 HT, Che Scientific Co., Hong Kong) at ambient temperature. This process afforded a yellow residue (329.0 g), which was purified by CC over silica gel (CH_3_Cl_3_–MeOH–H_2_O, 10:0:0 to 6:4:1—v/v) to give 4 fractions (Fractions A to D). Fraction C (5.8 g) was subjected to MCI CC (MeOH–H_2_O, 50:50 to 100:0—v/v) to obtain 9 fractions (Fractions C-1 to C-9). Fraction C-7 (1.0 g) was purified by CC over silica gel (PE-acetone, 10:0 to 5:5—v/v) to afford 11 fractions (Fractions C-7–1 to C-7–11). Fraction C-7–6 (267.0 mg) was finally purified by MCI CC (MeOH–H_2_O, 80:20 to 100:0) to yield compound **1** (12.0 mg) and compound **2** (17.0 mg) (Fig. 2).

### Kinase assay

An IKK *α* and *β* Assay/Inhibitor Screening Kit (CycLex, Nagano, Japan) and the IKK-*β* enzyme (2 units/200 µL, CycLex, Japan) were used to screen the IKK-*β* inhibitory activities of compounds **1** and **2**, as well as that of the reported IKK-*β* inhibitor berberine [16], at concentrations in the range of 1–100 µM according to the manufacturer’s protocol [21]. Briefly, the tested compounds were dissolved in dimethyl sulfoxide (DMSO) to obtain 10 mM stock solutions. The stock solutions were diluted at least ten-fold with kinase buffer to afford the sample solutions. Each sample solution (10 µL) or a blank control (10 µL of 10 % DMSO in kinase buffer) was added into a well pre-coated with a substrate corresponding to recombinant I*κ*B*α* on ice. The IKK-*β* enzyme (10 mUnits/µL) was diluted ten-fold with kinase buffer, and 10 µL of the diluted enzyme solution was added to each well. Each well was then treated with 80 µL of a freshly prepared adenosine triphosphate (ATP) solution (62.5 µM in kinase buffer). The total volume of each reaction was 100 µL containing the desired concentrations of 1, 10, 50 and 100 µM of the test compounds. A solution of 1 % DMSO in kinase buffer was used as a solvent control. The wells were subsequently incubated at 30 °C with gentle shaking at 90 rpm for 30 min. The reaction mixtures in each well were discarded, and then washed five times with 100 µL of 10X washing buffer containing 2 % Tween^®^-20. An anti-phospho-I*κ*B*α*-S32 monoclonal antibody (AS-2E8, 100 µL) was added to the wells, and the resulting mixtures were incubated at 25 °C with gentle shaking (90 rpm) for 30 min. After discarding the reaction mixtures and washing the wells five times with 10X washing buffer containing 2 % Tween^®^-20, each well was treated with 100 µL of a horseradish peroxidase (HRP) conjugated anti-mouse IgG solution, before being incubated under the same condition as those described the previous step for 30 min. All of the reaction mixtures were subsequently discarded and the well washed five times with 10X washing buffer containing 2 % Tween^®^-20 before being treated with tetramethylbenzidine (TMB, 100 µL) and incubated at 25 °C with shaking at 90 rpm for 15 min. The reactions were terminated by the addition of a 0.5 N solution of sulfuric acid (100 µL), followed by the agitation of the resulting mixtures on a shaker for 5 min at room temperature. Finally, the absorbance of the sample solutions was measured at a wavelength of 450 nm using a multimode reader (Infinite M200 PRO, Tecan, Männedorf, Switzerland). All of the samples were assayed in triplicate. The solvent control was regarded as 100 % phosphorylation of I*κ*B*α* by the IKK-*β* enzyme [21].

### Statistical analysis

The IKK-*β* activities of the compound-treated vials were normalized based on the activity of the solvent control, which was regarded as 100 % of the IKK-*β* activity. These values were then expressed as the mean ± standard deviation (SD). Differences between the results of each compound and berberine at all tested concentrations were performed by a two-tailed Student’s *t* test and subsequent Bonferroni correction of *P* values. In addition, IKK-*β* inhibitory activities between compounds **1** and **2** at all tested concentrations were also compared using a two-tailed Student’s *t* test in order to summarize the structure activity relationship. Differences were considered statistically significant for *P* < 0.05. Dose-dependency was determined visually by observing the trends of the data.

## Results

### Isolation and structural elucidation of compounds **1** and **2**

The methanolic extract of the air dried flowers of *C. gigantea* was subjected to a series of normal and reverse phase chromatographic purification process to obtain compounds **1** and **2** (Fig. 1). The molecular formula of compound **1** was determined to be C_28_H_42_O_3_ by HRMS in the positive ionization mode, which gave a molecular ion with an *m*/*z* value of 427.3186 for [M+H]^+^ (calcd for [M+H]^+^ 427.3207, with a measurement error of 4.9 ppm) (Additional file 1). HRMS analysis also revealed fragment ions with *m*/*z* values of 409.3087, 393.3134 and 375.3031, corresponding to [M−H_2_O+H]^+^, [M−H_2_O_2_+H]^+^ and [M−H_2_O_2_−H_2_O+H]^+^, respectively, which suggested the presence of hydroxyl and peroxy groups in this molecule [19, 22].

The ^1^H NMR spectrum of compound **1** (Additional file 2) contained signals consistent with the presence of six methyl groups [*δ*_H_ 1.09*, s*, H-19; *δ*_H_ 0.73*, s*, H-18; *δ*_H_ 1.00*, d* (*J* = 6.6 Hz), H-21; *δ*_H_ 0.91*, d* (*J* = 7.2 Hz), H-28; *δ*_H_ 0.83*, d* (*J* = 6.6 Hz), H-26 and *δ*_H_ 0.82*, d* (*J* = 7.2 Hz), H-27] and five olefinic protons [*δ*_H_ 5.42*, dd* (*J* = 6.0, 1.8 Hz), H-11; *δ*_H_ 6.28*, d* (*J* = 8.4 Hz), H-6; *δ*_H_ 6.59*, d* (*J* = 8.4 Hz), H-7; *δ*_H_ 5.16*, dd* (*J* = 15.0, 8.4 Hz), H-22 and *δ*_H_ 5.24*, dd* (*J* = 15.0, 7.8 Hz), H-23]. The coupling constant between H-6 and H-7 was around 8.4 Hz, which suggested that these two protons were coupled with each other in a *cis*-orientation, whereas the coupling pattern between H-22 and H-23 was consistent with these protons sitting in a *trans*-orientation based on their large coupling constant (15.0 Hz).

The ^13^C NMR spectrum of compound **1** (Additional file 3) contained 28 carbon signals (Table 1). Six of the carbon signals in the down field region of the spectrum were consistent with the presence of three double bonds (*δ*_C_ 135.4, C-6; *δ*_C_ 130.7, C-7; *δ*_C_142.5, C-9; *δ*_C_ 119.7, C-11; *δ*_C_ 135.1, C-22 and *δ*_C_ 132.4, C-23). Three oxygenated carbons (*δ*_C_ 82.7, C-5; *δ*_C_ 78.4, C-8 and *δ*_C_ 66.3, C-3) were also observed in the ^13^C NMR spectrum. The 19 remaining carbon signals were consistent with the presence of quaternary carbons and aliphatic carbons (e.g., methine, methylene and methyl carbons). Taken together with NMR data (Table 1) from the literature [19, 23], these data indicated that compound **1** was 9,11-dehydroergosterol peroxide.

**Table 1:** ^1^H and ^13^C NMR data of compounds **1** and **2** in CDCl_3_

Position	**1**		**2**	
	*δ*_C_	*δ*_H_ (*J* in Hz)	*δ*_C_	*δ*_H_ (*J* in Hz)
1	32.6		34.7	
2	30.6		29.6	
3	66.3	4.01 (*m*)	66.1	3.86 (*m*)
4	36.0		36.7	
5	82.7		82.3	
6	135.4	6.28 (*d*, 8.4)	135.2	6.19 (*d*, 8.4)
7	130.7	6.59 (*d*, 8.4)	130.6	6.45 (*d*, 8.4)
8	78.4		79.6	
9	142.5		51.0	
10	38.0		36.9	
11	119.7	5.42 (*dd*, 6.0, 1.8)	23.3	
12	41.2		39.3	
13	43.6		44.5	
14	48.2		51.6	
15	20.9		20.5	
16	28.6		28.6	
17	55.8		56.1	
18	13.0	0.73 (*s*)	12.8	0.76 (*brs*)
19	25.6	1.09 (*s*)	18.1	0.83 (*brs*)
20	39.9		39.7	
21	20.7	1.00 (*d*, 6.6)	20.8	0.94 (*d*, 6.6)
22	135.1	5.16 (*dd*, 15.0, 8.4)	135.5	5.09 (*dd*, 15.0, 8.4)
23	132.4	5.24 (*dd*, 15.0, 7.8)	132.3	5.17 (*dd*, 15.0, 7.8)
24	42.8		42.7	
25	33.1		33.0	
26	19.9	0.83 (*d*, 6.6)	19.6	0.76 (*d*, 6.6)
27	19.6	0.82 (*d*, 7.2)	19.9	0.78 (*d*, 6.6)
28	17.6	0.91 (*d*, 7.2)	17.5	0.86 (*d*, 6.6)

The HRMS analysis of compound **2** in the positive ion mode (Additional file 4) gave a molecular ion with an *m/z* value of 429.3346 for [M+H]^+^ (calcd for [M+H]^+^= 429.3363 with measurement error of 4.0 ppm). This result indicated that the mass of compound **2** was two mass units greater than that of compound **1**, suggesting that the molecular formula of compound **2** was C_28_H_44_O_3_. The fragmentation pattern in the mass spectrum of compound **2** was very similar to that of compound **1**. The detection of fragment ions with *m*/*z* values of 411.3239 [M−H_2_O+H]^+^, 395.3292 [M−H_2_O_2_+H]^+^ and 377.3191 [M−H_2_O_2_−H_2_O+H]^+^ indicated that compound **2** contained hydroxyl and peroxy groups [19].

The ^1^H NMR spectrum of compound **2** (Additional file 5) showed six methyl groups [*δ*_H_ 0.94*, d* (*J* = 6.6 Hz), H-21; *δ*_H_ 0.86*, d* (*J* = 6.6 Hz), H-28; *δ*_H_ 0.83*, brs*, H-19;*δ*_H_ 0.78*, d* (*J* = 6.6 Hz), H-27; *δ*_H_ 0.76*, d* (*J* = 6.6 Hz), H-26 and *δ*_H_ 0.76*, brs*, H-18] and four olefinic protons [*δ*_H_ 6.45, *d* (*J* = 8.4 Hz), H-7; *δ*_H_ 6.19*, d* (*J* = 8.4 Hz), H-6; *δ*_H_ 5.17, *dd* (*J* = 15.0, 7.8 Hz), H-23 and *δ*_H_ 5.09*, dd* (*J* = 15.0, 8.4 Hz), H-22].

The ^13^C NMR spectrum of compound **2** (Additional file 6) contained signals consistent with the presence of three oxygenated carbons (*δ*_C_ 82.3, C-5; *δ*_C_ 79.6, C-8 and *δ*_C_ 66.1, C-3) and two C=C double bonds in the down field region (*δ*_C_ 135.2, C-6; *δ*_C_ 130.6, C-7; *δ*_C_ 135.5, C-22 and *δ*_C_ 132.3, C-23), which were similar to those observed for compound **1**, except for the absence of one C=C double bond. Notably, these spectroscopic data were consistent with those reported in the literature for a known compound [19, 23, 24]. Compound **2** was therefore characterized as ergosterol peroxide.

### IKK-*β* inhibitory activity of compounds 1 and 2

IKK-*β*, which is a key subunit of IKK [17], is involved in the phosphorylation of I*κ*B*α* as part of the signaling pathway responsible for the transcription of NF-*κ*B [25]. The IKK-mediated phosphorylation of the Ser32 and Ser36 residues of cytoplasmic I*κ*B*α* leads to the poly-ubiquitination and subsequent proteasomal degradation of I*κ*B*α*, which results in the release of NF-*κ*B and its subsequent translocation to the nucleus [17, 18].

Compounds **1** and **2** have been reported to exhibit anti-cancer and anti-inflammatory effects through their ability to inhibit the NF-*κ*B signaling pathway [13–15]. The in vitro inhibitory effects of these two epidioxysterols towards IKK-*β* were evaluated in the current study using a CycLex IKK-*β* Assay/Inhibitor Screening Kit, which is a single-site, semi-quantitative immunoassay. Briefly, the IKK-*β* enzyme and the recombinant I*κ*B*α* substrate (pre-coated on the bottom of the wells) were incubated in the presence of ATP and various concentrations of compounds **1** and **2** in the wells of a 96-well plate at 30 °C for 30 min. The phosphorylated I*κ*B*α* substrate was detected using an AS-2E8 specific antibody, followed by a HRP-conjugated secondary antibody with subsequent color development using a TMB substrate. A dilute H_2_SO_4_ solution was used to stop the color development and the absorbance was read at 450 nm. The absorbance was directly related to the level of IKK-*β* activity.

**Fig. 3:**
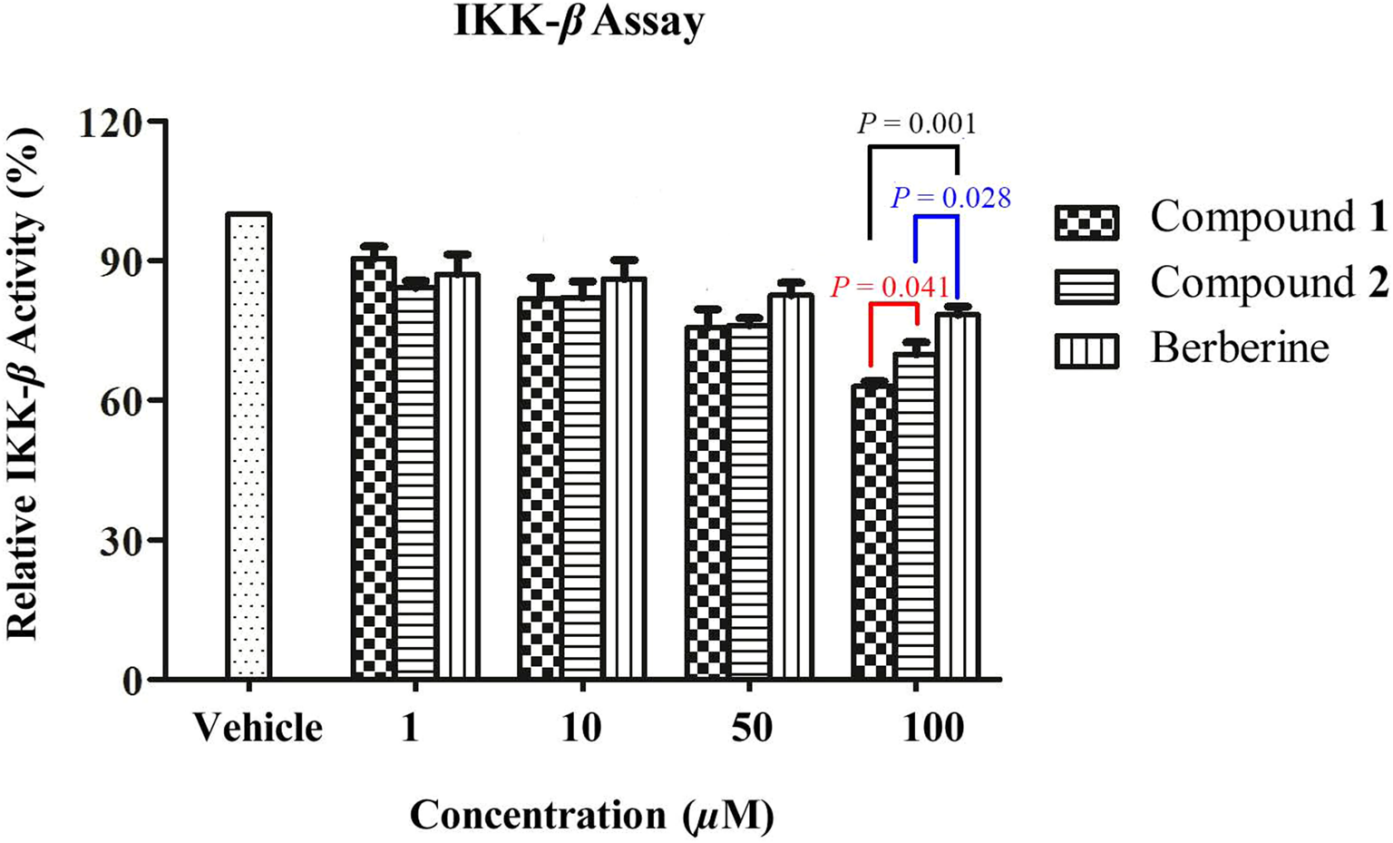
Relative IKK-*β* activities of compounds **1** and **2**. The results represent the mean ±SD from three independent experiments. The *P* values are shown as indicated

As shown in Fig. 3 and Table 2, compounds **1** and **2** exhibited dose-dependent inhibitory activity towards IKK-*β* in a similar manner to the control compound berberine. At concentrations in the range of 1–50 μM, the percentage inhibition values of compounds **1** and **2** towards IKK-*β* were in the ranges of 9.6–24.4 % and 15.8–24.0 %, respectively. In general, the inhibitory activities of the two epidioxysterols towards IKK-*β* were similar to that (13.0–17.4 %) of berberine. Interestingly, at a concentration of 100 μM, the inhibitory activities of compounds **1** and **2** towards IKK-*β* were 1.7 fold (*P* = 0.001) and 1.4 fold (*P* = 0.028), respectively, greater than that of berberine. Furthermore, compound **1** exhibited greater inhibitory activity towards IKK-*β* than compound **2** (*P* = 0.041) at the same concentration. However, the activities of these two compounds were much similar at the lower concentrations of 10 and 50 μM. These results therefore indicated that the C=C double bond between C-9 and C-11 in 9,11-dehydroergosterol peroxide could responsible for the enhanced activity of this compound towards IKK-*β* at the higher dose.

**Table 2:** In vitro IKK-*β* inhibitory activity (%) of compounds **1**, **2** and berberine

Concentration (µM)	IKK-*β* inhibitory activity (%)		
	Compound **1**	Compound **2**	Berberine
1	9.6 (2.7)	15.8 (1.5)	13.0 (4.3)
10	18.2 (4.5)	17.9 (3.5)	14.0 (4.2)
50	24.4 (3.9)	24.0 (1.7)	17.4 (2.7)
100	37.1 (1.1)	30.2 (2.6)	21.7 (1.8)

The results are mean (SD) from three independent experiments

## Discussion

A kinase assay was used to examine the inhibitory activities of compounds **1** and **2** towards IKK-*β* and the NF-*κ*B pathway. Berberine (10 µM) effectively attenuated the activity of IKK-*β* using an immunocomplex kinase assay method [16]. In contrast, the same concentration of berberine only inhibited the activity of IKK-*β* by 14 % in the current study, which suggested that the assay method used in the current study was not as sensitive as the immunocomplex kinase assay. Furthermore, compounds **1** and **2** exhibited greater inhibitory activities towards IKK-*β* than that of berberine at a high concentration of 100 μM. However, it remains unclear whether the inhibitory activity of these compounds was due to them binding directly to IKK-*β* in a similar manner to berberine. Further study will therefore be required to elucidate the mechanism of action of these compounds.

Our results revealed that compound **1** exhibited greater inhibitory activity towards IKK-*β* than compound **2** at 100 µM. Furthermore, the IC_50_ values of compounds **1** and **2** towards Hep 3B cancer cells were 39.2 and 45.3 µM, respectively [20]. The parallels between the IKK-*β* inhibitory activities and cytotoxicities of these compounds towards cancer cells indicated the existence of a structure activity relationship involving the double bond between C-9 and C-11. This double bond appeared to contribute to the IKK-*β* inhibitory activity and cytotoxicity of 9,11-dehydroergosterol peroxide (**1**) at the higher concentration of 100 µM. This result indicated that more planar ergosterol peroxide derivatives could show more potent inhibitory effects towards IKK-*β*. This structure activity relationship could therefore guide the future design and synthesis of more planar dehydrated derivatives based on these two epidioxysterols as stronger IKK-*β* inhibitors. Although the inhibitory activities of compounds **1** and **2** towards IKK-*β* were limited to the in vitro kinase assay in the present study, cell-based bioassays could be performed to determine whether the IKK-*β* enzyme is the direct target of these two epidioxysterols in the NF-*κ*B pathway.

This study provided the first example of the isolation of compounds **1** and **2** from a plant in the Asclepiadaceae family. Moreover, this is also the first demonstration the in vitro IKK-*β* inhibitory activities of ergosterol peroxide and its derivative.

## Conclusion

Two epidioxysterols, 9,11-dehydroergosterol peroxide (**1**) and ergosterol peroxide (**2**), were isolated from the flowers of *C. gigantea* and exhibited in vitro IKK-*β* inhibitory activities at concentrations in the range of 10–100 µM.

## Additional files

10.1186/s13020-016-0081-1 HRMS spectrum (positive mode) of compound **1**.
10.1186/s13020-016-0081-1 ^1^H NMR spectrum of compound **1**.
10.1186/s13020-016-0081-1 ^13^C NMR spectrum of compound **1**.
10.1186/s13020-016-0081-1 HRMS spectrum (positive mode) of compound **2**.
10.1186/s13020-016-0081-1 ^1^H NMR spectrum of compound **2**.
10.1186/s13020-016-0081-1 ^13^C NMR spectrum of compound **2**.

## Abbreviations

APCI: atmospheric pressure chemical ionization
ATP: adenosine triphosphate
AS-2E8: an anti-phospho-I*κ*B*α*-S32 monoclonal antibody
CC: column chromatographies
DMSO: dimethyl sulfoxide
Hep 3B: human hepatocellular carcinoma cells
HL60: human leukemia cells
HRMS: high resolution mass spectra
HRP: horseradish peroxidase
HT29: human colon adenocarcinoma cells
IKK: i*κ*B kinase
NF-*κ*B: nuclear factor kappa B
TMB: tetra-methylbenzidine
TOF: time-of-flight
