# Supplementary figures and images for: Inhibition of IKK-β by epidioxysterols from the flowers of Calotropis gigantea (Niu jiao gua)
Source: Chin Med. 2016 Mar 2;11:9. doi: 10.1186/s13020-016-0081-1 (PMC4774138; doi:10.1186/s13020-016-0081-1)

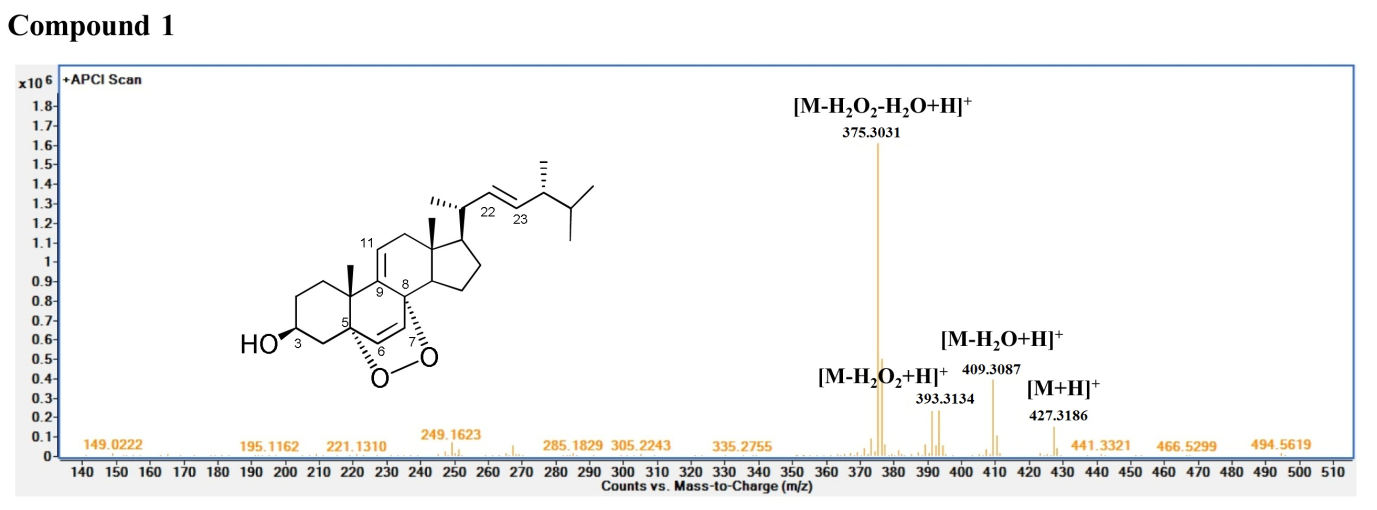

Supplement: Supplementary file 1 — 10.1186/s13020-016-0081-1 HRMS spectrum (positive mode) of compound 1. [file 13020_2016_81_MOESM1_ESM.docx]

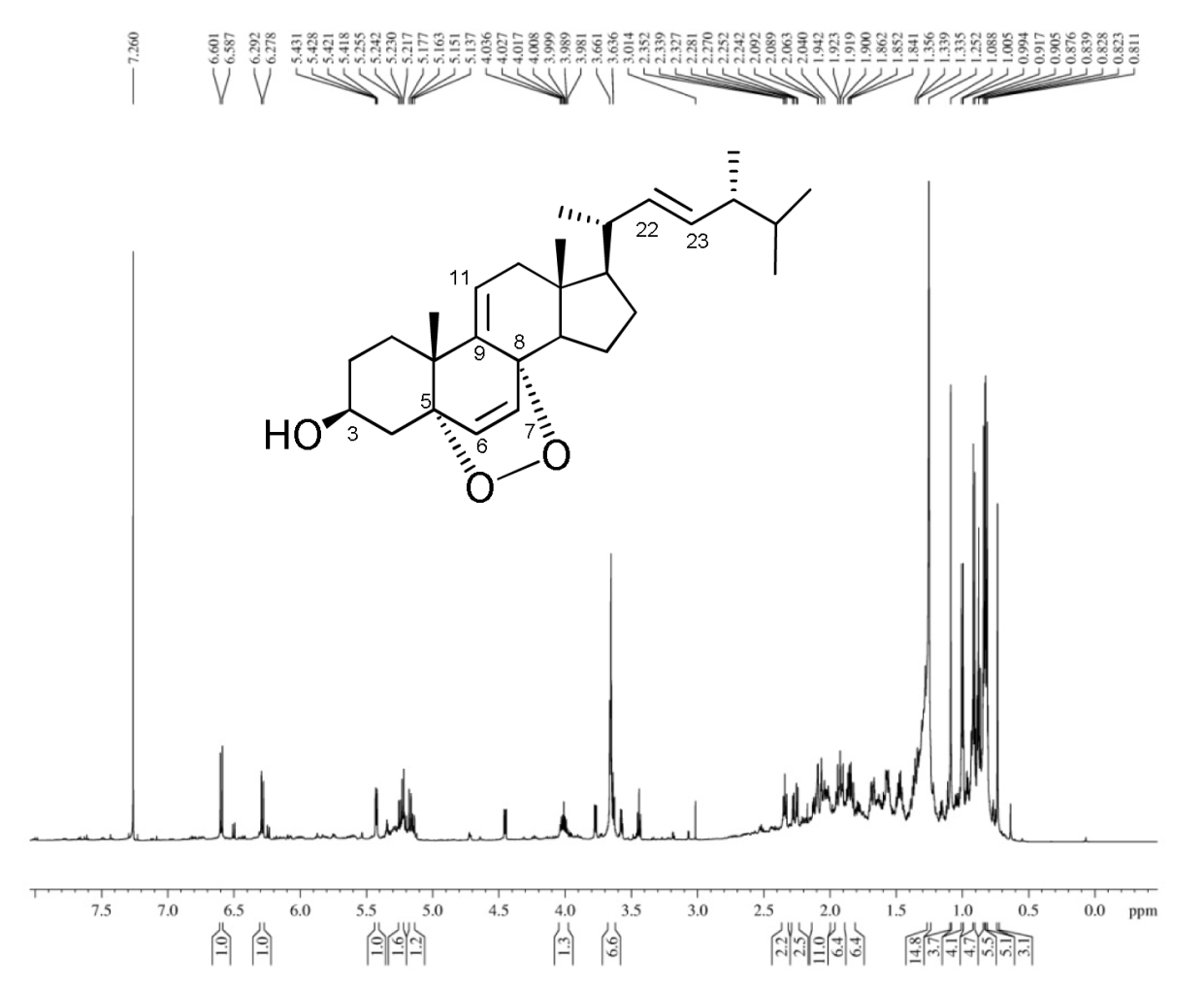

Supplement: Supplementary file 2 — 10.1186/s13020-016-0081-1 1H NMR spectrum of compound 1. [file 13020_2016_81_MOESM2_ESM.docx]

**
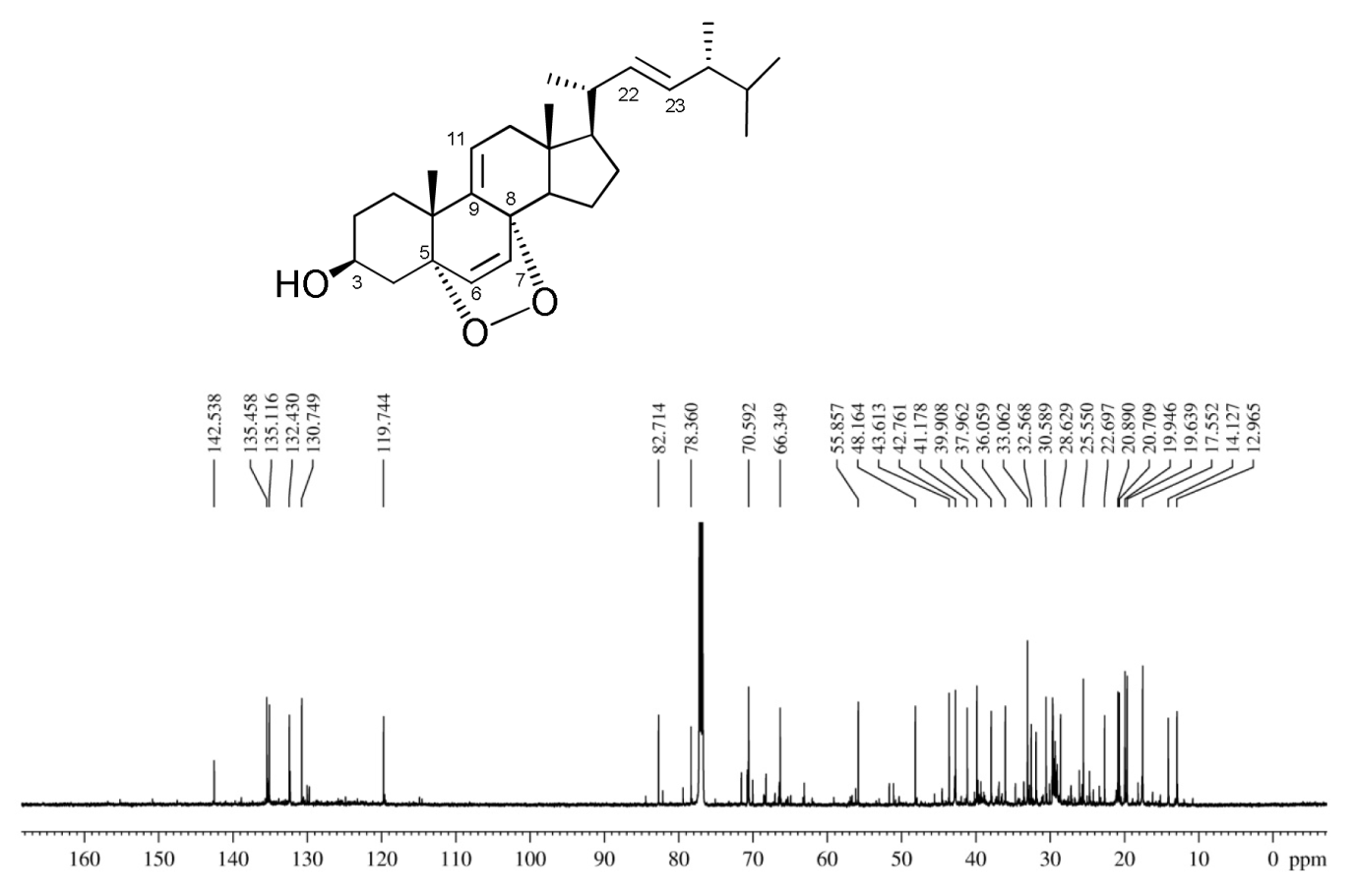
**

Supplement: Supplementary file 3 — 10.1186/s13020-016-0081-1 13C NMR spectrum of compound 1. [file 13020_2016_81_MOESM3_ESM.docx]

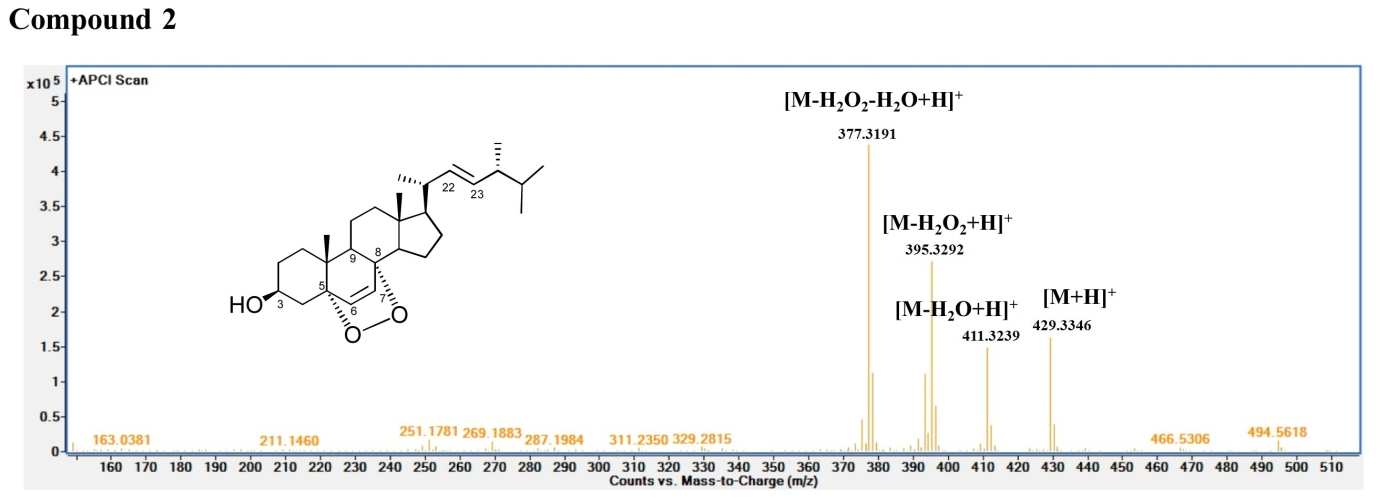

Supplement: Supplementary file 4 — 10.1186/s13020-016-0081-1 HRMS spectrum (positive mode) of compound 2. [file 13020_2016_81_MOESM4_ESM.docx]

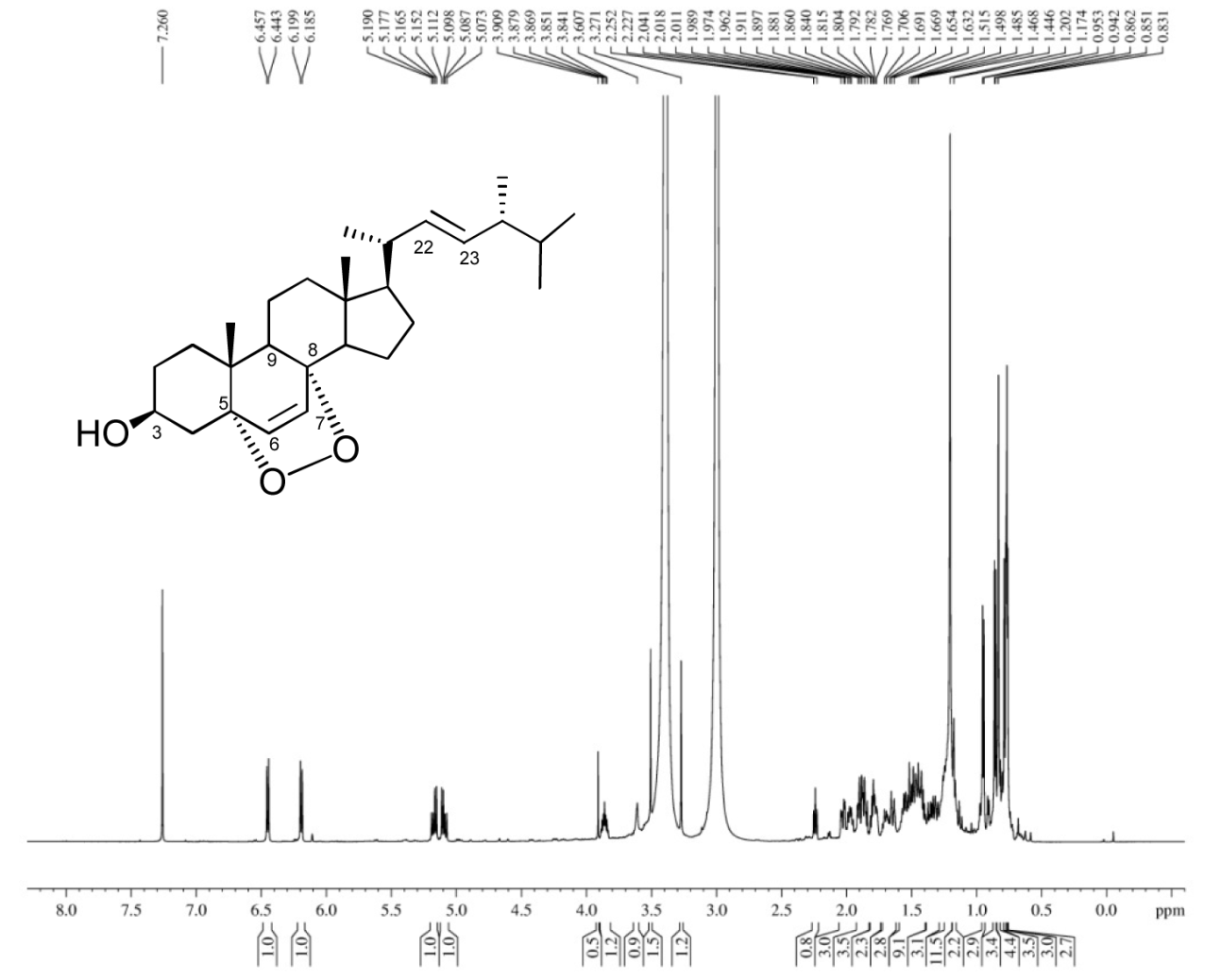

Supplement: Supplementary file 5 — 10.1186/s13020-016-0081-1 1H NMR spectrum of compound 2. [file 13020_2016_81_MOESM5_ESM.docx]

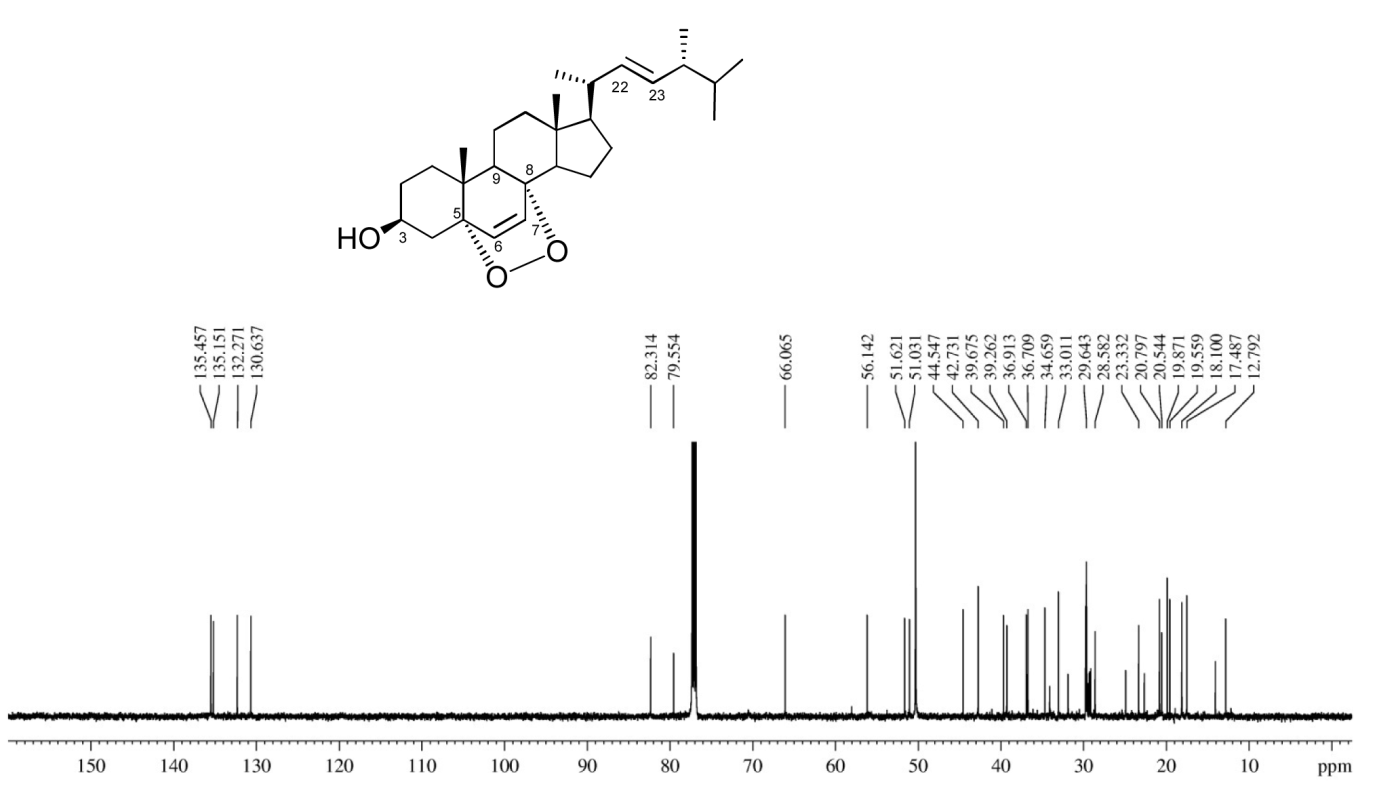

Supplement: Supplementary file 6 — 10.1186/s13020-016-0081-1 13C NMR spectrum of compound 2. [file 13020_2016_81_MOESM6_ESM.docx]
